# Nano-Anatase TiO_2_ for High Performance Optical Humidity Sensing on Chip

**DOI:** 10.3390/s16010039

**Published:** 2015-12-29

**Authors:** Mahdiar Ghadiry, Mehrdad Gholami, Lai Choon Kong, Chong Wu Yi, Harith Ahmad, Yatima Alias

**Affiliations:** 1 Photonics Research Center, University of Malaya, Kuala Lumpur 50603, Malaysia; laichoonkong@gmail.com (L.C.K.); WuYi@um.edu.my (C.W.Y.); Harith@um.edu.my (H.A.); 2 Department of Chemistry, Faculty of Science, University of Malaya, Kuala Lumpur 50603, Malaysia; gholami897@gmail.com (M.G.); Yatima@um.edu.my (Y.A.)

**Keywords:** humidity sensing, nano-anatase TiO_2_, waveguide, evanescence field, optic sensor

## Abstract

An on-chip optical humidity sensor using Nano-anatase TiO_2_ coating is presented here. The coating material was prepared so that the result is in solution form, making the fabrication process quick and simple. Then, the solution was effortlessly spin-coated on an SU8 straight channel waveguide. Investigating the sensitivity and performance (response time) of the device revealed a great linearity in the wide range (35% to 98%) of relative humidity (RH). In addition, a variation of more than 14 dB in transmitted optical power was observed, with a response time of only ~0.7 s. The effect of coating concentration and UV treatment was examined on the performance and repeatability of the sensor. Interesting observations were found, and the attributed mechanisms were described. In addition, the proposed sensor was extensively compared with other state-of-the-art proposed counterparts from the literature and remarkable advantages were found. Since a high sensitivity of ~0.21 dB/%RH and high dynamic performances were demonstrated, this sensor is proposed for use in biomedical applications.

## 1. Introduction

Humidity monitoring is a necessary activity in many industry fields [1]. Measurement of humidity is of great importance in chemical and medical industries such as respiration monitoring [2,3]. In addition, humidity sensing is essential in civil and aerospace engineering. It is frequently monitored in giant structures such as planes and bridges to control the possible risk of leakage due to corrosion [4]. Furthermore, it affects product quality and the health of workers in the food industry [5]. Therefore, it has been the focus of research for decades and a great variety of humidity sensors; resistive, capacitive, optical and thermal conductivity have been proposed and examined [6].

In a humidity sensor, low response time, high sensitivity, repeatability, reversibility and noise immunity are required [7,8]. Low power loss (attenuation) and a high bandwidth of optical networks using fibre optics increase the possibility of transmitting large amounts of data over kilometre-long distances [9]. In addition, the simple geometry and small size of optical sensors make the implementation of light weight sensors possible such that they can be embedded in construction material and medical probes simply [2]. Finally, the dynamic range and sensitivity of optical sensors can be substantially greater than that of conventional electrochemical sensors. All of these promising properties make optical sensors a suitable choice for applications where electronic or acoustic ones are either inappropriate or not recommended [10].

Furthermore, in situations where humidity changes rapidly, sensors with low response and recovery times are needed. Such applications include process control in industry, meteorology and a variety of other medical applications such as human respiration monitoring [11,12]. This shows the need for fast, highly sensitive and low-cost humidity sensors. 

Moreover, fabrication simplicity and low cost are also of importance in humidity sensors. Humidity sensors based on organic coatings are particularly attractive because the sensing material is prepared using a simple solution, which is in contrast with inorganic materials, which demand expensive preparation methods such as vapour-liquid-solid growth and chemical vapour deposition (CVD) [11]. However, organic sensors generally suffer from instability in terms of performance because they show high response and recovery times. In general, using nanoscale coating results in an increase in the sensitivity and response time of the sensors due to the high surface area of the coating material compared to its thickness [13,14]. However, the fabrication process is normally difficult and requires expensive machines. Therefore, there is need for a simple, fast, and efficient method to develop sensors [15].

Here, an optical humidity sensor using nano-anatase TiO_2_ particles is reported and compared with its state-of-the-art counterparts. For fabrication, a new approach is employed to prepare TiO_2_ nanoparticles in solution form, making the fabrication low-cost and simple. The advantages of the proposed sensor are its high sensitivity, low response and recovery times, and simple fabrication process. 

## 2. Experimental 

### 2.1. Preparation of the Materials 

Titanium isopropoxide and urea were purchased from Aldrich, and acetylacetone was purchased from Merck. The aqueous solutions were prepared using deionized water with a resistivity of ∼18 MΩ·cm^−1^. All of the glass apparatuses were kept in 1.0 mol·L^−1^ nitric acid when they were not in use.

### 2.2. Synthesis of Nano-Anatase TiO_2_ Powder 

Initially, 2 mL of aetylacetone was mixed with 2 mL titanium isopropoxide in a 10 mL measuring cylinder. The prepared solution was then mixed with 40 mL of absolute ethanol in a beaker via vigorous stirring at room temperature. Subsequently, we added the mixed solution and a solution containing 10 mL of 5% (W/V) of urea dropwise in a beaker via stirring at room temperature. The final solution was pale yellow in color with a pH value of 5.6. After vigorous stirring of this solution for one hour, the whole mixture was transferred into a 120-mL Teflon-lined stainless steel autoclave for 18 h at 150 °C and cooled down naturally. The produced solution was centrifuged with deionized water and absolute ethanol a number of times. This residue was dried for 3 h at 80 °C. The powder was ground to obtain a fine powder, which was used for the preparation of nano-Anatase TiO_2_ solutions of different concentrations. In this experiment, solutions of 0.01% (NAT1), 0.02% (NAT2), 0.04% (NAT3) and 0.08% (NAT4) (W/V) of nano-Anatase TiO_2_ were prepared from the resulting solid powder.

### 2.3. Field Emission Scanning Electron Microscopy (FESEM)

**Figure 1:**
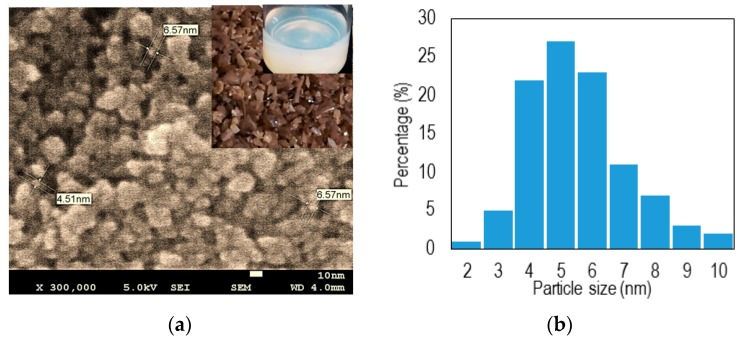
(**a**) The FESEM images of nano-anatase titanium dioxide (inset: the powder and its solution form); (**b**) Normalized distribution of the particle sizes.

Morphological and structural analysis of the samples was performed by field emission scanning electron microscopy (FESEM) using a Hitachi SU8000 (Tokyo, Japan) microscope. Figure 1a shows the FESEM of synthesized powder, the powder and the solution (insets). According to our size distribution analysis, (see Figure 1b), most particle sizes had a diameter less than 10 nm and the maximum concentration was seen at 5 nm. 

### 2.4. Device Fabrication 

**Figure 2:**
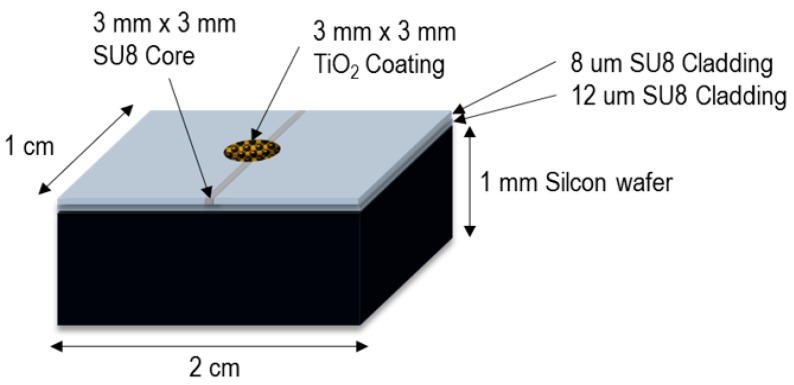
Dimention and structure of the fabricated device.

A waveguide was fabricated on a SiO_2_ wafer as the substrate. This polymer-based waveguide sensor consisted of 2 layers, a undercladding layer and a core layer. Both of them were made from an SU-8 2010 polymer with 100% and 70% concentrations. By controlling the time of curing, a difference layer with a difference reflective index was created. The undercladding layer with a thickness of 12 μm and refractive index of 1.569 was spin-coated on the substrate and cured at 95 °C for a soft-bake of 2 min and a post-bake of 40 s, respectively. As for the core layer, another SU-8 polymer layer, with a thickness of 8 μm and a refractive index of 1.572 measured at 1550 nm, was spin-coated on the undercladding layer and patterned by means of contact photolithography. Again, the sample was subjected to another curing process at 95 °C for a soft-bake of 30 min and a post-bake of 10 s, respectively. Then, the TiO_2_ solution was drop-casted on the waveguide, and the sample was kept at room temperature until dried. Then, it was washed with DI water, and UV was applied. The dimensions are stated in Figure 2. For more detail, please refer to [16]. 

### 2.5. Humidity Sensing Measurement

**Figure 3:**
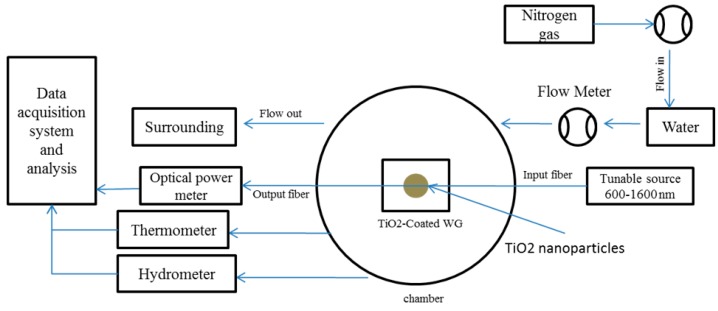
Experimental setup to control humidity and temperature in the chamber and measure the power loss variation at different RHs.

The experimental setup is illustrated in Figure 3. A gas pipe supplying controlled humidity vapor was located about 1 cm above the device. Humid nitrogen gas was formed by bubbling dry nitrogen gas through water before flowing out from the gas tube. The response time of the sensor to humid air (~90% RH) was measured by a photodiode and by employing a mechanical shutter between the gas source and the sensing region to control the exposure intervals. The fiber was bonded to the waveguide in order to minimize the error and power fluctuations of the output due to misalignments.

**Figure 4:**
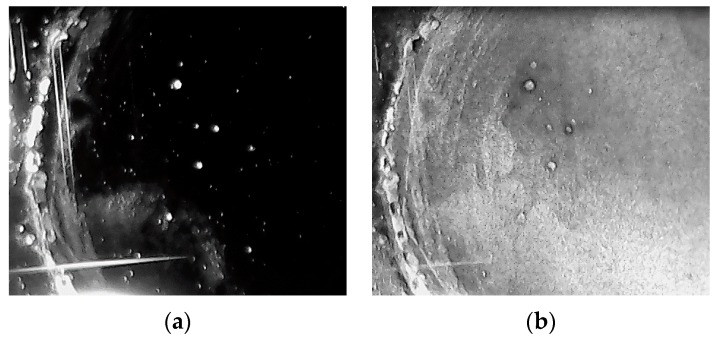
TiO_2_ coating on SiO_2_ substrate at room condition (**a**) and exposed to 90% humidity (**b**).

The top view of the fabricated sample was taken when it was exposed to dry (Figure 4a) and humid air (Figure 4b), respectively. As can be seen, the coating is almost transparent when it is not exposed to water vapor. 

## 3. Results and Discussion

The sensing mechanism is quite simple. The propagation of the electromagnetic field inside the waveguide core is not completely confined. A fraction of the field, called the evanescent field, escapes the core; it penetrates into, and propagates inside, the outer layer (coating). The guided field (inside the core) and the evanescent field are connected by a condition of continuity of the electromagnetic field on the border between the core and the coating. Thus, the guided field will be influenced by any variation in the evanescent field [17]. 

Generally, increasing RH causes the coating refractive index (RI) to increase due to cladding water absorption [6]. This significantly changes the boundary condition at the guide-cladding interface, which reduces the beam confinement in the core (guide). Consequently, the output power loss through the cladded guide decreases. 

The field strength is dependent on the input strength, the initial refractive index ratio of core/coating, and the thickness of the coating. As a result, coating material concentration (thickness) plays an significant role in optical properties of the sensor [18]. Therefore, several concentrations (thickness) of the material were prepared and applied to the device, and the sensitivity and performance (response and recovery times) are reported in Table 1. It can be seen that, by increasing the concentration, the sensitivity increases while the performance decreases. 

**Table 1:** Sensitivity and performance of the proposed device using several concentration of TiO_2_ as coating.

Concentration	Thickness	Sensitivity	Response Time	Recovery Time
	(µm)	(dB/%RH)	(s)	(s)
NAT1	0.21	0.021	0.22	0.31
NAT2	0.53	0.12	0.61	0.75
NAT3	0.74	0.21	0.72	0.95
NAT4	1.01	0.414	1.12	1.61

Using a layer with a thickness much less than that of the evanescence field penetration depth (NAT1) resulted in a low number of immobilized particles available for interacting with the evanescence wave. When the humidity increases, a low number of water molecules can be diffused in the TiO_2_ layer, since a low number of nanoparticles interact with them. Thus, low power variation was observed, and low sensitivity was experienced. On the other hand, based on the same argument, the association and dissociation of water molecules were fast. 

However, in the thick layer of coating, many water molecules were trapped (association), and the change in the refractive index was causing high sensitivity. On the other hand, the association and dissociation processes took time, which caused the response and recovery time to increase. In addition, the hysteresis of the device increased due to non-dissociated molecules trapped in the coating layer.

**Figure 5:**
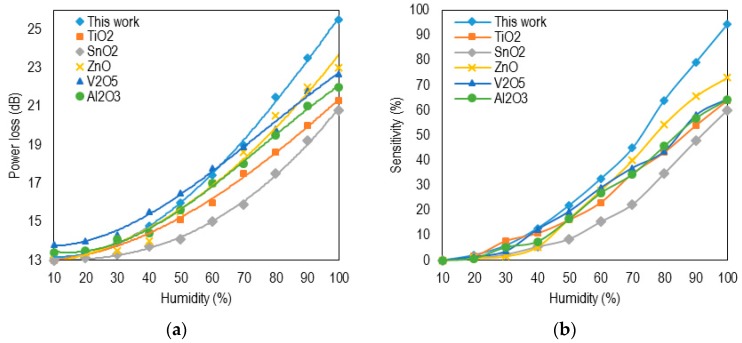
Profile of power loss (**a**) and sensitivity (**b**) of the proposed sensor compared to other sensors using different coating materials. There are two regions with low sensitivity in the range from 10% to 35% and high sensitivity in the range of 35% to 98%. More information regarding preparation of other materials could be found in [6].

Since both sensitivity and performance was the focus of this study, the third sensor using NAT3 was then investigated. The power loss profile of the proposed sensor with respect to relative humidity (RH) was compared with other comparable devices using various coating materials, shown in Figure 5a. The linear profile for the proposed sensor is seen for RH in the range from 35% to 98%. The range of variation in power loss was seen to be large from 13.6 to 25.5 dB for RH, ranging from 30% to 98%. As a result, high sensitivity was experienced, shown in Figure 5b. In this figure, the y-axis *(L_0_* *− L_RH_)/L_RH_* represents the ratio of the change in the power loss, whereas *L_0_* and *L_RH_* are the power loss of the device at 10% and 98% RH, respectively [19]. The power fluctuation rate due to noise was less than 0.1 dB, which is not significant compared to the wide range of variation in power loss. As a result, a high accuracy of up to 1% RH was observed. 

Low sensitivity was observed at low RH (10% to 35%). In this range, water molecules may be dominantly absorbed at the surface, which causes a small amount of light presented at the cladding-air to be absorbed. This does not affect the guide-cladding coupling as much as the cladding. However, in a higher RH range (35% to 90%), monolayers of water molecules will be adsorbed onto the walls of the holes in the cladding, which may change the real part of cladding RI. This results in significant radiation and affects the transverse guiding in the coating. Although the core RI dominantly has the real part in the first region initially, the imaginary part might contribute as well; thus, the sensitivity increases. Stewart *et al.* [9] have reported a similar mechanism with an increasing concentration. In the current case, water vapor was used instead of gas molecules; hence, the identical phenomena was expected. Above 90%, multilayer absorption of water molecules was experienced, which resulted in a saturation and sensitivity decrease.

**Figure 6:**
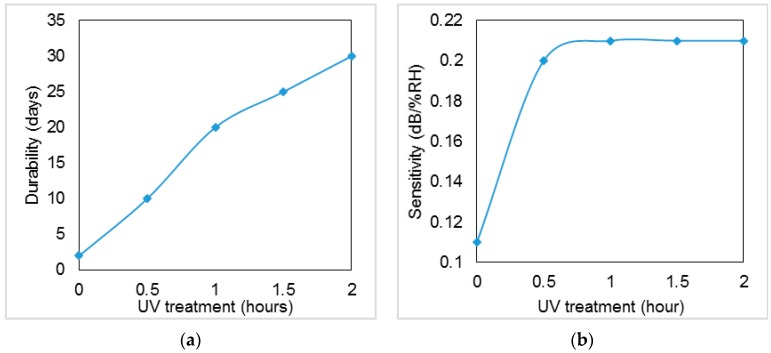
UV exposure effect on sensitivity (**a**) and stability or repeatability (**b**) of the proposed sensor.

The effect of UV exposure on the device sensitivity was studied by varying UV treatment from 0 to 2 h. As it can be seen in Figure 6a,b, by increasing the UV exposure time, the sensitivity and durability of the sensor increases respectively. Durability indicates how long the sensor can be used to maintain the original properties. Figure 6a shows that, by using UV exposure for 2 h, the fabricated sensor can be used for 30 days with no significant accuracy loss. It is worth mentioning that we did not continue the tests for more than 30 days.

The attributed mechanisms will now be explained. The water molecule contact angle (CA) is generally used to evaluate the surface wettability, which is defined as the angle between the tangent line of the liquid phase at the interface of the solid-liquid-gas phases and the solid surface [20]. The CA of TiO_2_ is initially about several tenth of degrees, depending on the surface roughness. As a result of exposing the surface to UV light, the CA decreases to almost zero degree (0°), meaning that the water molecules spread out flat, and the surface becomes highly hydrophilic. 

In addition, it has already been reported that the material hardness is affected by UV light irradiation [21]. These outcomes suggests that the extremely hydrophilic surface was subjected to compressive stress caused by the expansion of the surface volume [21]. 

**Figure 7:**
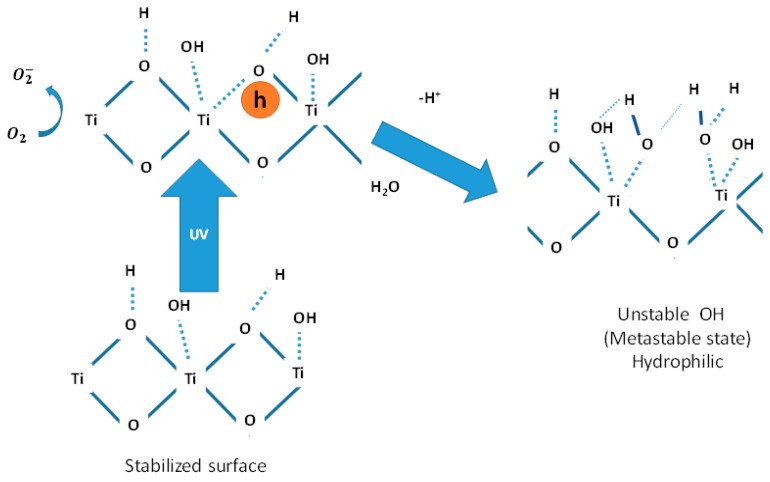
Mechanism of surface wettability of TiO_2_ film under UV light irradiation.

It is believed that when TiO_2_ is irradiated with UV light, the electron-hole pairs are generated, and the photo-generated holes resulting in the bulk of the TiO_2_ are trapped at lattice oxygen sites [20,21]. Most trapped holes react with the adsorbed water, producing OH radicals as shown in Figure 7. Nevertheless, a small portion of the trapped holes might react with TiO_2_ itself through the coordination with water molecules at the titanium site and break the bond between the lattice titanium and oxygen ions. To compensate the charge, the coordinated water molecules release protons and produce new OH groups, causing an increase in the population of OH groups at the surface, resulting in the enhancement of their hydrophilic properties.

**Figure 8:**
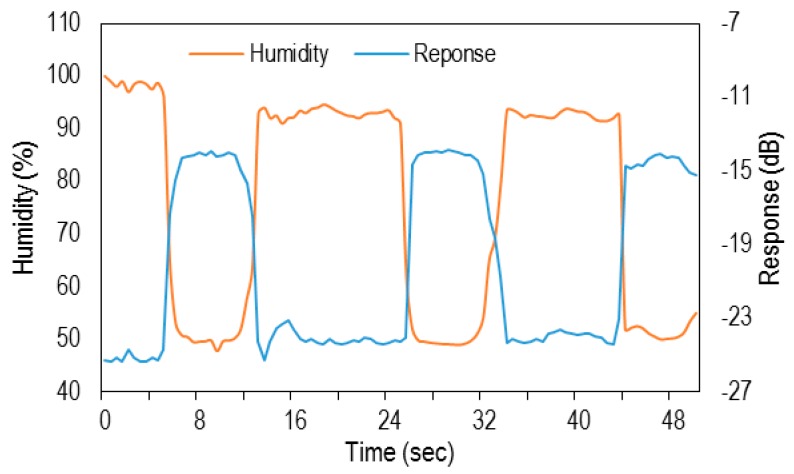
Observed response and recovery behavior of the humidity sensor for a cyclic humidity perturbation.

The device performance was then measured by disrupting the input dry and humid air mixture for certain time intervals. The results are plotted in Figure 8. For the third sensor (NAT3), the response and recovery time of 0.72 and 0.95 s, respectively, are measured. The fast response of the sensors can be credited to the fast diffusion of water molecules into the cladding, and the slower recovery is because of the slow desorption process. 

**Figure 9:**
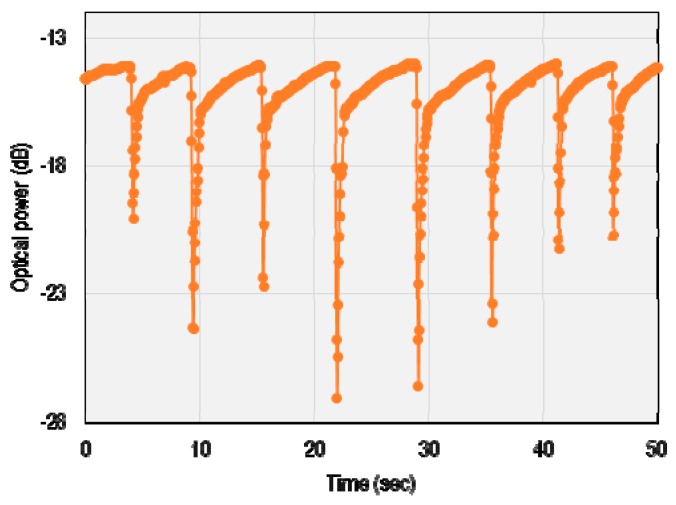
Sensor response to breathing from 10 cm distance with humidity raning from 50% to 100%.

In order to test the response time in more detail, specific data were recorded (see Figure 9). In this experiment, breath pulses with humidity ranging from 50% to 100% were expelled over the sample from a distance of almost 10 cm. A visional analysis of the results shows great reproducibility of the sensor. It is worth mentioning that the amount of humidity and timing in this experiment was not exactly controlled. 

**Figure 10:**
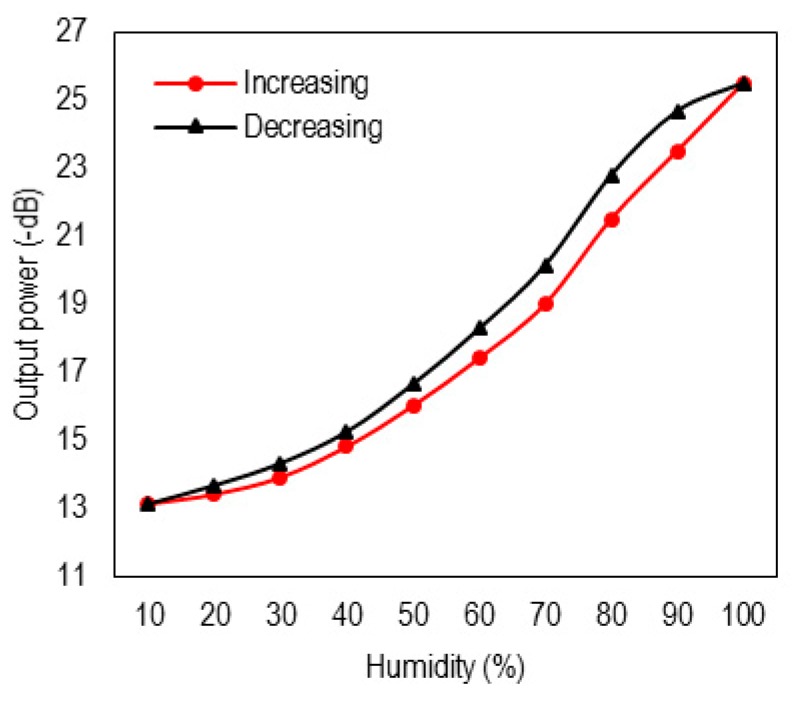
Response of the RH optical sensor when the humidity is decreased and increased in the range of 10%–98%.

As can be seen, successive rising and falling cycles of moisture show almost the same results within the experimental error values. However, to measure exact hysteresis values of the device, it was placed in the chamber again, and the output was measured for increasing and decreasing cycles. Figure 10 shows that the measured hysteresis is as low as ~6% at the worst case, which is due to the quick response time of the sensor—faster than the variation rate of the moisture inside the chamber.

For the purpose of giving a complete comparison, the proposed sensor is compared with other sensors using various methods and substrates, including single mode fiber waveguide, (SMF), long period fiber grating (LPG), fiber brag grating (FBG) and plastic-clad silica fiber (PCS) and cladding materials including Polyvinyl alcohol film, TiO_2_ film, gelatin, hydrogel, polyimide, a TiO_2_ nanoparticles, a ZnO nanoparticles, SiO_2_ nanoparticles, graphene oxide, and co-polyaniline. The result of the comparison is shown in Table 2. It is seen that the proposed sensor has 0.21 dB/RH, which is quite a high sensitivity over a wide range of RH and a very low response time compared to others. There are sensors with higher sensitivity than that of this work, but the range and/or their performance is not acceptable. In addition, our work uses coating material in solution form, making the fabrication process very easy and fast, while other compared devices use difficult processes. Furthermore, we investigated the sensor response to several gases such as CO, CO_2_, butane, nitrogen and oxygen, and no significant power variation was observed. As reported in the literature, a low thickness of coating, in range of a nanometer, is normally used for gas sensing [22], while the thickness we used is in the range of micrometers. Furthermore, the coating here is not functionalized and optimized [23] for gas sensing, and not much interaction can therefore be seen between TiO_2_ and gas molecules.

**Table 2:** Comparison of reported sensor with other proposed sensors using different coatings and methods.

Reference	Coating Material	Substrate	Range	Sensitivity	Resp. Time
This work	Nano-anatasTiO_2_	SU8 WG	35%–95%	0.21 dB/%RH	0.72 s
Lim *et al.* [24]	Graphene oxide	SU8 WG	60%–100%	0.53 dB/%RH	0.95 s
Herrero *et al.* [25]	TiO_2_ film	SMF	0%–15%	0.49 dB/%RH	-
Aneesh *et al.* [26]	TiO_2_ nanoparticles	PCS fiber	24%*–*95%	27 mV/%RH	>1 s
Corres *et al.* [7]	SiO_2_ nanoparticles	SMF	40%*–*98%	0.12 dB/%RH	0.15 s
Aneesh *et al.* [27]	ZnO nanoparticles	PCS	4%*–*96%	0:0012RH-1	>1 s
Yeo *et al.* [28]	Polyimide	FBG	23%*–*97%	5.6 dB/%RH	>5 min
Gaston *et al.* [29]	Polyvinyl alcohol film	SMF	70%*–*90%	0.51 dB/%RH	40 s
Tan *et al.* [30]	Gelatin	LPG	90%*–*99%	1.2 dB/%RH	-
Liu *et al.* [31]	Hydrogel	LPG	38%*–*100%	0.2 nm/%RH	-
Vijayan *et al.* [32]	Co-polyaniline	Optical fiber	20%*–*95%	6 mV/%RH	~1 min

## 4. Conclusions 

An optical humidity sensor is presented here based on the effect of the evanescent field in an anatase TiO_2_ nanoparticle coating. Our approach to preparing the active material resulted in TiO_2_ in solution form, which makes the fabrication process quick, easy and cheap. The sensor sensitivity and performance were extensively investigated at different humidity conditions. It was observed that the device response is almost linear over a wide dynamic range (35%–98% RH) with a high sensitivity of around 0.21 dB/%RH. In addition, the effect of several parameters such as coating thickness and the UV treatment time were studied in terms of repeatability, performance and sensitivity of the device, and the attributed mechanisms were explained. It was revealed that, by increasing the thickness, the sensitivity increases while the performance decreases. In addition, we showed that exposing UV results in an increase in sensitivity and repeatability. With a high sensitivity and a low response time of ~0.7 s for humidification and 0.95 s for desiccation, the device is most suitable for real time human respiration monitoring applications. It is believed that this approach could be used for other applications as well, such as PH meters, temperature sensors and glucose concentration sensing.
